# Assessment of different manufacturing techniques for the production of bioartificial scaffolds as soft organ transplant substitutes

**DOI:** 10.3389/fbioe.2023.1186351

**Published:** 2023-06-27

**Authors:** Silvia Pisani, Valeria Mauri, Erika Negrello, Simone Mauramati, Gianluca Alaimo, Ferdinando Auricchio, Marco Benazzo, Rossella Dorati, Ida Genta, Bice Conti, Virginia Valeria Ferretti, Annalisa De Silvestri, Andrea Pietrabissa, Stefania Marconi

**Affiliations:** ^1^ Department of Otorhinolaryngology, Fondazione IRCCS Policlinico San Matteo, Pavia, Italy; ^2^ SC General Surgery 2, Fondazione IRCCS Policlinico San Matteo, Pavia, Italy; ^3^ Department of Civil Engineering and Architecture, University of Pavia, Pavia, Italy; ^4^ Department of Clinical, Surgical, Diagnostic and Pediatric Sciences, University of Pavia, Pavia, Italy; ^5^ Department of Drug Sciences, University of Pavia, Pavia, Italy; ^6^ SSD Biostatistica e Clinical Trial Center, Fondazione IRCCS Policlinico San Matteo, Pavia, Italy; ^7^ Department of Surgery, University of Pavia, Pavia, Italy; ^8^ Fondazione IRCCS Policlinico San Matteo, Pavia, Italy

**Keywords:** bioartificial scaffolds, tissue engineering, 3D printing, electrospinning, transplantology, organ transplant, soft tissue regeneration

## Abstract

**Introduction:** The problem of organs’ shortage for transplantation is widely known: different manufacturing techniques such as Solvent casting, Electrospinning and 3D Printing were considered to produce bioartificial scaffolds for tissue engineering purposes and possible transplantation substitutes. The advantages of manufacturing techniques’ combination to develop hybrid scaffolds with increased performing properties was also evaluated.

**Methods:** Scaffolds were produced using poly-L-lactide-co-caprolactone (PLA-PCL) copolymer and characterized for their morphological, biological, and mechanical features.

**Results:** Hybrid scaffolds showed the best properties in terms of viability (>100%) and cell adhesion. Furthermore, their mechanical properties were found to be comparable with the reference values for soft tissues (range 1–10 MPa).

**Discussion:** The created hybrid scaffolds pave the way for the future development of more complex systems capable of supporting, from a morphological, mechanical, and biological standpoint, the physiological needs of the tissues/organs to be transplanted.

## Graphical Abstract

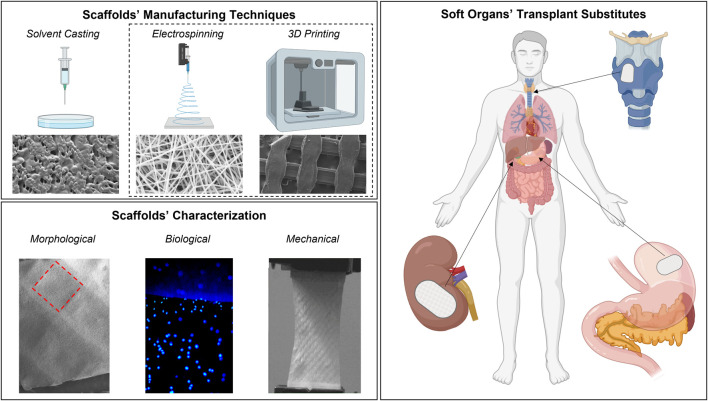

## 1 Introduction

Organ failure is still a leading cause of mortality worldwide, despite advances in surgical and transplantation techniques. The allogeneic transplant is still one of the most used strategies (more than 34,285 solid organ transplants were performed in the EU in 2019) (Vanholder et al., 2021). However, it has a series of problems such as the shortage of organs and donor cross-matching (Beyar, 2011). Indeed, it was reported by The World Health Organization (WHO) that only 10% of the worldwide need for organ transplantation is being met (Keeping Kidneys, 2012). To overcome the scarcity of donors, xenotransplantation techniques are being used (commonly from pigs) (Cooper et al., 2016; Zhang et al., 2019). However, this technique still features many limitations, including the most important one, a severe immune reaction concomitant with coagulation dysregulation and chronic inflammation; these adverse effects can nullify the transplant surgery and compromise a patient’s life (Zhou et al., 2022).

Bioartificial organs (BOs) are emerging as a valid alternative to traditional organ transplantation able to obviate the lack of donors and avoid adverse reactions (Wang, 2019). Essentially, a BO is an engineered three-dimensional (3D) scaffold implanted or integrated into the human body, able to interact with surrounding living tissues with the aim of replacing an organ, promoting regeneration, and restoring its original functionality (Abu-Faraj, 2012; Ren and Ott, 2014).

The overarching vision is to provide better approaches for the development of bioartificial scaffold (BSs)-guided tissues to be applied to personalized therapy, organ replacement, or reconstruction as well as to *ex vivo* screening among treatment options for different human diseases (Oksdath et al., 2018). For BOs development, three-dimensional scaffolds alone are not enough for a correct and complete replacement of the damaged tissues, but it is preferred as the co-presence of suitable cells is able to make the repair process faster and more effective. In fact, the BSs geometry and architecture must mimic physiological tissue/organ and promote proper cell adhesion and proliferation to guarantee tissue/organ integration and functionality (Causa et al., 2007). As an example, fibroblast cells are widely used in tissue engineering thanks to their heterogeneous presence in numerous tissues. Depending on the anatomical region of origin, it has been demonstrated that fibroblasts have different gene expression profiles (Wong et al., 2007). Among them, dermal fibroblasts were demonstrated to have several functions such as increasing ECM components synthesis and deposition to promote proliferation and migration in response to stimuli (cytokines) and exhibiting autocrine and paracrine interaction (Stunova and Vistejnova, 2018). Therefore, the harmony and synergy between the 3D scaffold and the sown cells are fundamental to guarantee suitable chemical-physical, mechanical, and biological characteristics to mimic the physiological properties of the tissue to be replaced (Sultana, 2018; Okamoto, 2019). Crucial aspects must be considered to provide the right support for cell adhesion, growth, and/or differentiation on BSs; these include the choice of the polymer, the manufacturing technique, the geometry, and the consequent scaffolds’ mechanical properties (Bacakova et al., 2011; Eltom et al., 2019). As for polymers, it is essential to choose biocompatible and non-toxic polymers such as Poly-lactic acid (PLA), Poly-caprolactone (PCL), Poly-lactic-co-glycolic acid (PLGA), or polyurethane (Pedersen et al., 2022). Many of these polymers are also biodegradable, therefore ensuring the tissue reformation and avoiding the generation of toxic degradation products; the main advantage of these polymers is that they do not have to be surgically removed once the tissue is grown and therefore do not require further interventions for the patient (Arif et al., 2019; Nikolova and Chavali, 2019).

Scaffold geometry provides biophysical signals that trigger cells’ nucleus response (i.e., the regulation of gene expression) and modulates cells’ behavior and functionality (Han et al., 2022). Geometrical parameters are also crucial to mimic the characteristics of the tissue/organ to be replaced and to improve cell proliferation: different cellular lines require different scaffold geometries and porosity percentages according to the function they have to perform (Chantarapanich et al., 2012; Loh and Choong, 2013).

Various techniques for the BSs production have been implemented over the years, starting from the simplest ones-such as solvent casting (SC)-up to the more advanced ones, such as electrospinning (ES) and 3D printing (3DP) (Murphy and Mikos, 2007; Wang, 2019; Morelli et al., 2022; Pien et al., 2022). SC technique is a scaffold manufacturing method that begins with the dissolution of a polymer in an organic solvent which afterwards hardens by exploiting solvent evaporation; a dry solid polymer scaffold with a porous network is left behind, although it is difficult to handle the desired pore shape and pore inter-connectivity of these types of scaffolds (Johnson et al., 2010; Dorati et al., 2017). ES is a fibers’ fabrication technique which allows to obtain scaffolds consisting of a network of submicrometric-sized fibers. The collected electrospun fibers exhibit suitable properties for biomedical applications such as high surface area-to-volume ratio, small pore size, high porosity, and a structure able to mimic ECM (Pisani et al., 2018). More recently, 3DP technologies have emerged as versatile techniques to produce components to be used for a wide range of purposes such as anatomical models, surgical planning, training and simulation, medical devices, prosthetics prototyping, and regenerative medicine (Conti and Marconi, 2019). With Computer-Aided Design (CAD) programs, it is possible to define the geometry, the infill percentage, and the structure porosity according to 3D printers’ resolution limits.

The purpose of the present work was to produce and characterize BSs manufactured through three different manufacturing techniques (SC, EL, and 3DP). Moreover, the advantages of combining EL and 3DP for the development of hybrid scaffolds (HS) useful for soft tissue replacement was evaluated. The combination of ES and 3DP technologies is already present with some examples in the literature. The important aspect highlighted in this work is that for the same used base material, obtained scaffolds have different properties due precisely to the manufacturing technique. Therefore, one of the main goals is to underline how the production technique has a strong influence on the final scaffolds’ properties (both mechanical and biological).

BSs were made up by the same biopolymer (poli-L-lactide-co-caprolactone—PLA-PCL 70:30 ratio) that showed PLA prevalence with a suitable degradation rate (about 8 weeks *in vivo*) commensurate with the rate of tissue regeneration, while the PCL component gives plasticity. Moreover, PLA-PCL was chosen for its known bio-properties, such as biocompatibility and biodegradability, and because it has already been widely characterized in previous works by the authors, demonstrating excellent properties of soft tissue regeneration (Pisani et al., 2018; Pisani et al., 2020; Pisani et al., 2021). In the literature, the combination of Electrospinning (ES) and Fused Filament Fabrication (FFF) to produce copolymer PLA-PCL scaffolds usually refers to a hybrid scaffold made of a layer of 3D-printed PLA and a layer of electrospun PCL (or *vice versa*) (Pensa et al., 2019; Smith and Mele, 2021); on the contrary, here HS where both ES and 3DP layers are made with the same PLA-PCL copolymer are proposed. The resulting BSs were compared in terms of morphological, mechanical, and biological properties and the influence of manufacturing technique on the final features was evaluated and compared. This proof of concept lays the foundations for the subsequent development of complete bioartificial soft body organs with the aim of providing replacement models for transplantation.

## 2 Materials and methods

### 2.1 Scaffold manufacturing techniques

The SC manufacturing technique was used to prepare PLA-PCL (Resomer LC 703 S—Mw 160,000 Da-Evonik Nutrition & Care GmbH, Evonik Industries AG Relling-hauser Straße 1–11, 45128 Essen, Germany) film-scaffold. The copolymer was solubilized in 1-4 dioxane since this organic solvent has a boiling point of +101°C and a melting point of +12°C. These features limit the evaporation of the solvent during the preparation phase, ensure complete freezing of the solvent during the cooling phase of the system, and guarantee its complete sublimation during freeze-drying, facilitating the removal of the solvent from the finished product. The polymeric solution (10% w/v) was placed in a glass vial and subjected to magnetic stirring (1 h at RT) until completely dissolved. Once a solution was obtained, it was sonicated for 15 min (Sonica, Ultrasonic Cleaner. Soltec, Italy) to remove any air bubbles, which in the subsequent dripping phase could give rise to the formation of morphological heterogeneities, affecting the scaffold formation and reproducibility. Using a glass syringe (Gastight Syringes^®^/1 mL, Model 1001 LT SYR, Hamilton), 800 μL of the PLA-PCL solution in 1,4-dioxane was dropped into a Teflon mold 5 × 5 cm. After the dripping phase, the mold was frozen at −25°C for 5 h, followed by the freeze-drying process (Lyophilizer Lio-5P, Cinquepascal, Italy) at −48°C and 0.4 mbar for 12 h, to eliminate all the solvent residues and favor the formation of the dry polymeric film.

The same PLA-PCL copolymer was used to produce electrospun nanofibers. A 20% w/v polymeric solution in Methylene Chloride (MC) and N, N-Dimethylformamide (DMF) solvent blend 70:30 ratio was electrospun following parameters optimized in previous works (Pisani et al., 2018; Conti and Marconi, 2019). The setup parameters were voltage (30 kV), flowrate (0.5 ml/h), needle-collector distance (15 cm), temperature (25 ± 3°C), and relative humidity (RH% = 30 ± 4%). Electrospinning apparatus NANON-01A equipped with a dehumidifier (MEEC instruments, MP, Pioltello, Italy) was used to produce electrospun polymeric fibers. PLA-PCL fibers were collected after 30 min spinning time using a metal plate collector (14 × 25 cm).

3DP scaffolds were produced using a PLA-PCL (70:30) copolymer filament (TreeD Filaments^®^ company) through Fused Filament Fabrication (FFF) 3D printing technology. The filament had a calibrated diameter of 1.75 mm and was extruded - without any non-natural additive—using a LeapFrog HS^®^ (Dutch LeapFrog Group). The printer was equipped with two extruders with nozzles of diameter 0.35 mm and a heated bed - with a printing area of 230 × 270 × 200 mm—that can reach a temperature of 90°C. The two extruders were equipped with a resistance heating them up to 250°C. The printing head was also equipped with two fans to cool the filament right before the extrusion chamber, to keep it rigid and able to push the heated polymer out of the nozzle.

The printer was instructed on how to deploy the material through a specific set of instructions, formatted according to the G-code language, which included information on the path, temperatures of the nozzles and the bed, printing speed, flow rate, etc. The G-code was computed thanks to the so-called slicing software, which started from virtual 3D geometry to be produced and generated the set of instructions to drive the production. In the present work, Ultimaker Cura 4.11.0 software was employed to generate the final G-code. To set-up the optimal printing strategy for the PLA-PCL copolymer, a preliminary investigation of printing parameters was required. On the one hand, PLA filament is one of the most common materials used in FFF processes and its printing parameters are therefore already well-known. On the other hand, little information is available in the literature about PCL printing parameters (printing temperatures (Darling and Sun, 2004), extrusion rates, and requirement of a ventilation system cooling off specimens during printing (Temple et al., 2014)), being less employed in FFF machine. Usually the two filaments (PLA and PCL) are printed separately, from two different nozzles, which are then combined layer-by-layer on the printing bed (Cheng et al., 2021; Espinosa and Moroni, 2021). In this work, the involved filament is unique and already includes both materials. PLA and PCL FFF printing parameters are widely known; on the contrary, the copolymer filament PLA-PCL 70:30 printing parameters had to be deeply studied and optimized for the specific purposes. Moreover, the production of a copolymer PLA-PCL filament is not a trivial task, due to the difficulty of producing a calibrated and regular filament with a diameter of 1.75 mm, starting from separate PLA and PCL granules. A change in PLA-PCL ratio would lead to different printing parameters, together with the possible use of various additives to facilitate the FFF scaffolds’ production. The use of a copolymer filament allowed us to characterize—from the biological and mechanical viewpoints - patches made of the exact same material but produced through both standard (SC and ES) and innovative (3DP) manufacturing techniques.

Accordingly, preliminary tests were conducted to define optimal PLA-PCL copolymer printing parameters, starting from basic parameters—such as bed and extrusion temperatures—and then more advanced options—such as speeds, flow rate, extrusion width, retraction speed, and length. Different printing parameters could influence the mechanical properties of the final FFF sample (Chaitat et al., 2022); considering the specific involved cells and patches’ geometry, the infill percentage parameter was considered as one of the most crucial aspects that could affect the final patches’ biological and mechanical properties and was involved for further investigation and analyses. Rectangular 3DP samples (10 × 20 × 0.4–0.5 mm) with three different infill percentages, namely, 20%, 50%, and 100%, were then produced. The main printing parameters for PLA-PCL patches production are listed in Table 1.

**TABLE 1:** Main slicing parameters according to Ultimaker Cura nomenclature.

Slicing parameter (ultimaker cura)	Value
Layer Height	0.20 mm
Extrusion Width	0.35 mm
Wall Thickness	0.80 mm
Top Layers	4
Bottom Layers	4
Top/Bottom Pattern	lines
Infill Pattern	grid
Infill Overlap Percentage	10%
Flowrate	100%
Printing Temperature	200°C
Printing Temperature (Initial Layer)	200°C
Build Plate Temperature	80°C
Build Plate Temperature (Initial Layer)	80°C
Infill Speed	60 mm/s
Wall Speed	30 mm/s
Outer Wall Speed	30 mm/s
Inner Wall Speed	60 mm/s
Top/Bottom Speed	30 mm/s
Enable Retraction	True
Retract at Layer Change	False
Retraction Distance	6.50 mm
Retraction Speed	25 mm/s
Build Plate Adhesion Type	skirt
Brim Width	8 mm
Fan Speed	100%

After a preliminary testing on SC, ES, and 3DP scaffolds respectively, the obtained results showed that ES and 3DP techniques allowed the production of the most promising scaffolds. Consequently, the combination of these two technologies was also considered and tested for HS manufacturing.

HS were aimed to combine the advantages of the two production techniques: on the one hand, 3DP produces very complex and defined geometries and has high process reproducibility, while the electrospun nanofibers allow to obtain a network resembling the native extracellular matrix, thus ensuring better cell adhesion and proliferation. Using the same parameters cited above, 20% w/v PLA-PCL fibers were electrospun for 30 min on 3DP PLA-PCL scaffolds (20%, 50% and 100% infill) to achieve a composite HS. The number of fibers deposited on 3DP scaffolds was evaluated gravimetrically on the dry scaffolds using analytical balance (BCE64-1S, Sartorius AG, Göttingen German).

### 2.2 Morphological analysis

Zeiss EVO MA10 apparatus (Carl Zeiss, Oberkochen, Germany) was used to perform SEM analyses on polymeric SC, ES, 3DP, and HS scaffolds to evaluate electrospun fibers’ dimensions and scaffolds porosity (%) per unit surface area. Analyses were performed at the following magnifications: 1.00 kX and 2.50 kX. The resulting images were processed by ImageJ software (an open-source image processing program designed for scientific multidimensional images) (Hotaling et al., 2015).

### 2.3 Gel permeation chromatography (GPC)

The chromatograph system consists of a guard column (Phenogel 10E 4˚A µm, 300 × 7.8 mm, Phenomenex, Milan, Italy) and three connected Ultrastyragel columns (7.7 × 250 mm each one with pores diameters of 104 ˚A, 103 ˚A and 500 ˚A), a pump (Varian 9,010), an infrared (IR) detector (Prostra 355 RI; Varian), and software used to process the data relating to MW (Galaxie Workstation, ver.1.8 Single-Instrument, Varian). Samples for GPC analysis were prepared by dissolving scaffolds (SC, ES and 3DP) in tetrahydrofuran (THF) at a concentration of 1–2 mg/mL. THF solutions were filtered through a 0.45 mm filter (Millipore, Massachusset, United States) and injected in the GPC apparatus; the GPC eluent was tetrahydrofuran at a flow rate of 1 mL/min. The resulting data of molecular weight (MW), molecular number (Mn), and polydispersity index (PDI) are expressed as an average of five parallel samples.

### 2.4 Mechanical characterization

Scaffolds’ mechanical characterization was performed through uniaxial tensile tests using an MTS Insight Testing Systems (MTS System Corporation). Tests were carried out at 10 mm/min crosshead speed using a 250N load cell. Testing parameters were selected according to the ASTM D638 standard, considered the most suitable for the materials under examination. Geometrical parameters—namely, specimens’ length (L), thickness (T), and width (W)—of the different types of scaffolds are reported in Table 2.

**TABLE 2:** Physical dimensions of patches produced with the different techniques.

	SC	ES	3DP	HS
L [mm]	25 ± 2	25 ± 1	20 ± 0.2	40 ± 2
T [mm]	2 ± 1	0.15 ± 0.03	0.4 ± 0.1	0.6 ± 0.2
W [mm]	15 ± 2	6 ± 1	10 ± 0.2	15 ± 0.5

Scaffolds’ stiffness was retrieved through linear interpolation of the initial part of stress-strain curves, using least square method (LSM) defined as the Young’s modulus (E). Reliability was assessed computing the maximum R2 factor of the linear interpolation of σ-ε curves for each sample (≥0.95).

Given the different size and thickness of the samples made with different techniques, results of mechanical characterization have been normalized to obtain comparable data.

### 2.5 Cell seeding and viability

Scaffolds’ cytocompatibility and cell adhesion were evaluated for SC, ES, 3DP, and HS 48 h after cells sowing, as defined by standard ISO 10993-5 (ISO 10993-5). Human Normal Dermal Fibroblast (HNDF) passage 5 (P.5) were used (100.000 cell/scaffold) for scaffolds cellularization. Scaffolds were fixed in CellCrown™ (CellCrown™ 12 NX Scaffdex, Tampere, Finland) supports, suitable for 12-multiwell, and sterilized before cell seeding. CellCrown™ was used to avoid samples floating in the cell culture medium which can cause cells to fall off the scaffold during cellularization. Scaffolds’ sanitization was performed by dipping scaffolds in an 85% v/v EtOH solution for 20 min followed by 15 min in a 70% v/v EtOH solution. Scaffolds were then washed with sterile PBS supplemented with 2% penicillin/streptomycin (P/S) and left under a laminar hood with UV irradiation overnight. A protocol for scaffolds’ sterilization was already used in previous work demonstrating that no scaffolds were damaged (Pisani et al., 2021).

Engineered scaffolds were maintained in cell culture medium (DMEM 10%v/v Fetal Bovine Serum) at 37°C and 5% CO_2_ for 48 h before analysis. The cell viability percentage was evaluated using underwent 3-(4,5-dymethiltiazol-2-y)-2,5 diphenyltetrazolium bromide (MTT) (Merck KGaA, Darmstadt, Germany) test. Cell culture media was removed from samples and the engineered scaffold was washed twice with PBS (pH 7.4). Subsequently, 300 μL of MTT solution (5 mg/mL in PBS) was added to each scaffold sample and fresh PBS was added to guarantee the total immersion of the samples. The scaffolds were maintained for 2 h and 30 min of incubation at 37°C and 5% of CO_2_, and then MTT solution was removed paying attention not to suck precipitated formazan salts.

The engineered scaffolds were withdrawn and transferred into a vial to be solubilized with 1 mL THF under magnetic stirring for 1 h to completely dissolve the polymeric matrix and lyse cellular membranes of cells adhered that gave a purple coloring proportional to cell viability. Instead, 12-multiwell bottoms (from where scaffolds were withdrawn) were treated with 1 mL DMSO and left under soaking for 1 h to lyse cellular membranes of cells that did not adhere to scaffolds. HNDF (100.000 cell/bottom) was used as control, treated with MTT solution for 2 h and 30 min, washed, and then solubilized with 1 mL DMSO. The solutions obtained by dissolution of the engineered scaffolds, from 12-multiwell bottoms and HNDF control, were spectrophotometrically analyzed at 570 nm wavelength in a quartz cell (6705 UV/Vis Spectrophotometer—Single cell holder JENWAY) to obtain Abs values and correlate them to cell viability.

DAPI (4′,6-diamidino-2-phenylindole) (Thermo Fisher Scientific Inc., Waltham, Massachusetts, US) is a fluorescent nuclear staining useful for highlighting the nuclei of the cells and detect cells on the scaffold surface. After 48 h incubation, scaffolds were washed with PBS and 0,4% v/v glutaraldehyde solution was added and left for 10 min to fix the adhered cells. Scaffolds were washed twice with PBS and TRITON X 0,1% v/v was added and left for 10 min in order to permeabilize the cell membranes. A further washing step with PBS was performed and scaffolds were treated with DAPI 300 nM solution over-night at 4°C. The samples were analyzed with fluorescence microscope (Leica DM IL LED with ebq 50 ac-L) and images obtained were processed by ImageJ software.

Cell visualization on scaffolds’ surfaces was performed using SEM analysis. After 48 h incubation, scaffolds were washed with PBS and dehydrated using ethanol (EtOH) solutions at increasing concentrations (30% v/v, 70% v/v, 80% v/v, and 100% v/v), for 10 min each passage. Samples were then washed with a 50:50 mixture of dry EtOH 100% and hexamethyldisilane (HDMS) for 20 min. Before SEM analysis, samples were left under a laminar hood to remove solvent residues and then made conductive with surface deposition of a thin layer of gold in an Argon atmosphere. SEM images were further processed by ImageJ software to characterize engineered scaffolds.

### 2.6 Statistical analysis

The results collected in this experimental study were presented as mean, standard deviation (SD), and 95% Confidence Interval (95%CI). All experiments were carried out in triplicate, unless otherwise stated. To take into account the correlation between technical triplicates (non-independence between observations), we fitted clustered regression models, using robust Sandwich estimator for the calculation of standard errors. The comparison of polymer molecular weight and molecular number for SC, ES, and 3DP techniques compared to the raw materials was evaluated by t-test for paired data.

Statistical analyses were performed using Stata 17 (StataCorp. 2021. Stata Statistical Software: Release 17. College Station, TX: StataCorp LLC). A *p*-value less than 0.05 was considered statistically significant.

## 3 Results

### 3.1 Morphological analysis

Results in terms of scaffolds’ weight, manufacturing time, and surface porosity resulting from the three different manufacturing techniques are reported in Table 3.

**TABLE 3:** SC, EL, 3DP, and HS scaffold information obtained after the manufacturing process.

Scaffold manufacturing technique	PLA-PCL 70:30	Production time	Surface porosity	Pore surface area
	**[mg]**	**[min]**	**[%]**	[mm^2^ **]**
SC	80 ± 5.32	1440	0.24 ± 0.05	0.00092 ± 0.00015
ES	50 ± 3.23	30	3.94 ± 1.12	0.00472 ± 0.0021
3DP 20% infill	120 ± 0.25	5	38.41 ± 4.45	2.3 ± 0.3
3DP 50% infill	230 ± 0.15	7	11.7 ± 2.42	0.2 ± 0.03
3DP 100% infill	350 ± 3.30	15	-	-
HS 20% infill	150 ± 0.41	5 + 30	4.14 ± 1.06	0.085 ± 0.1
HS 50% infill	250 ± 5.05	7 + 30	6.92 ± 1.02	0.069 ± 0.04
HS 100% infill	380 ± 1.50	15 + 30	8.28 ± 1.31	0.1399 ± 0.1

SC scaffolds showed a lower porosity compared to the ES ones, with an average pore size of 0.92 ± 0.15 μm^2^ and 4.72 ± 2.1 μm^2^ respectively.

**FIGURE 1:**
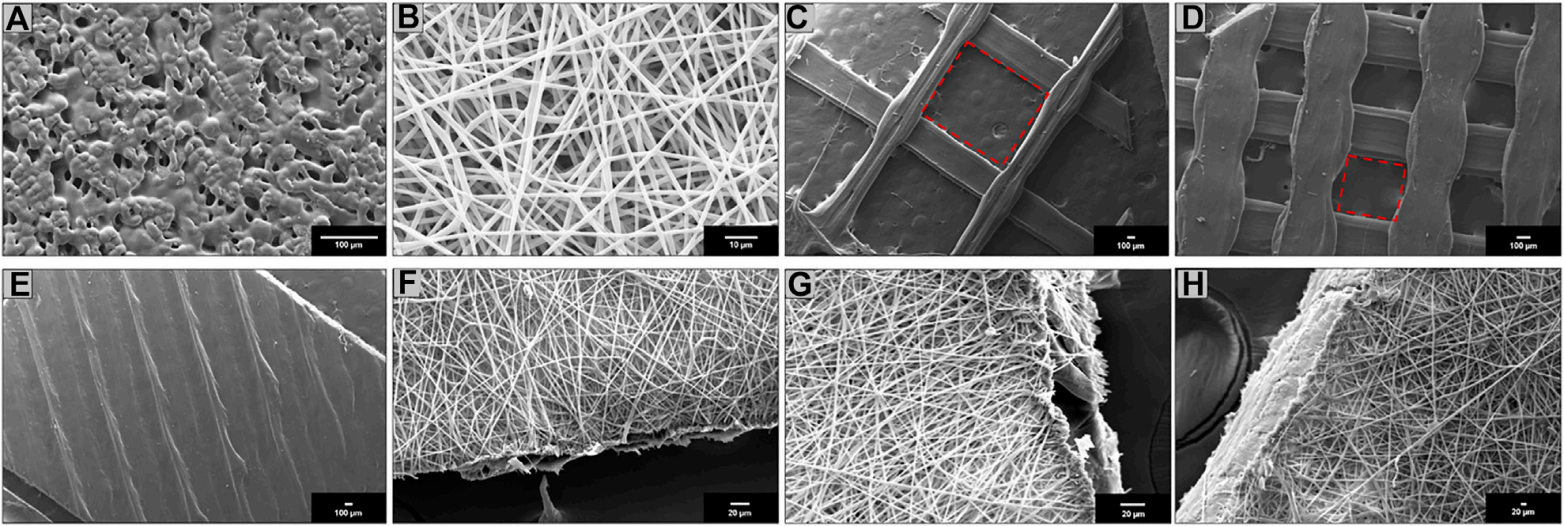
Scanning electron microscopy (SEM) of **(A)** SC; **(B)** ES; **(C)** 3DP 20% infill; **(D)** 3DP 50% infill; **(E)** 3DP 100% infill; **(F)** HS 20% infill; **(G)** HS 50% infill; and **(H)** HS 100% infill PLA-PCL 70:30 scaffolds.

To calculate the porosity of the 3DP scaffolds, the area of the square voids left by the filaments’ deposition was computed (Figures 1C,D). The porosity and average pore size progressively decreased from the lowest to the highest infill percentage; 3DP 100% infill resulted full of printed materials, i.e., 0 porosity. The single 3DP filament composing the 3DP scaffolds was in all cases pore-free and smooth.

HSs showed homogeneous fibers covering on 3DP scaffolds with an average weight increase of 25 ± 4.62 mg compared to the respective 3DP scaffold. The weight increase was comparable for all HS taking as reference their corresponding 3DP scaffolds. As concerns scaffolds’ porosity percentage and pore surface area, ES layer was considered.

SEM images were obtained for all scaffolds. SC scaffolds (Figure 1A) have shown undefined geometry and rough surface with some pores of variable size and random position generated by solvent sublimation during the manufacturing process. ES scaffolds (Figure 1B) are characterized by the presence of homogeneous nanofibers (745 ± 23 nm) forming an interconnected network with a high surface area and porosity. 3DP scaffolds obtained with three different infill, 20% (Figure 1C), 50% (Figure 1D), and 100% (Figure 1E), have shown polymeric filaments with a full, smooth, and non-porous structure; the porosity of the scaffold is controlled by the distance between the deposited filaments.

HS showed complete coverage and adhesion between the 3D scaffolds and electrospun fibers at all infills: 20% (Figure 1F), 50% (Figure 1G), and 100% (Figure 1H). Moreover, fibers electrospun on the 3DP scaffold maintained nanometer size range (775 ± 31 nm).

### 3.2 Gel permeation chromatography (GPC)

**FIGURE 2:**
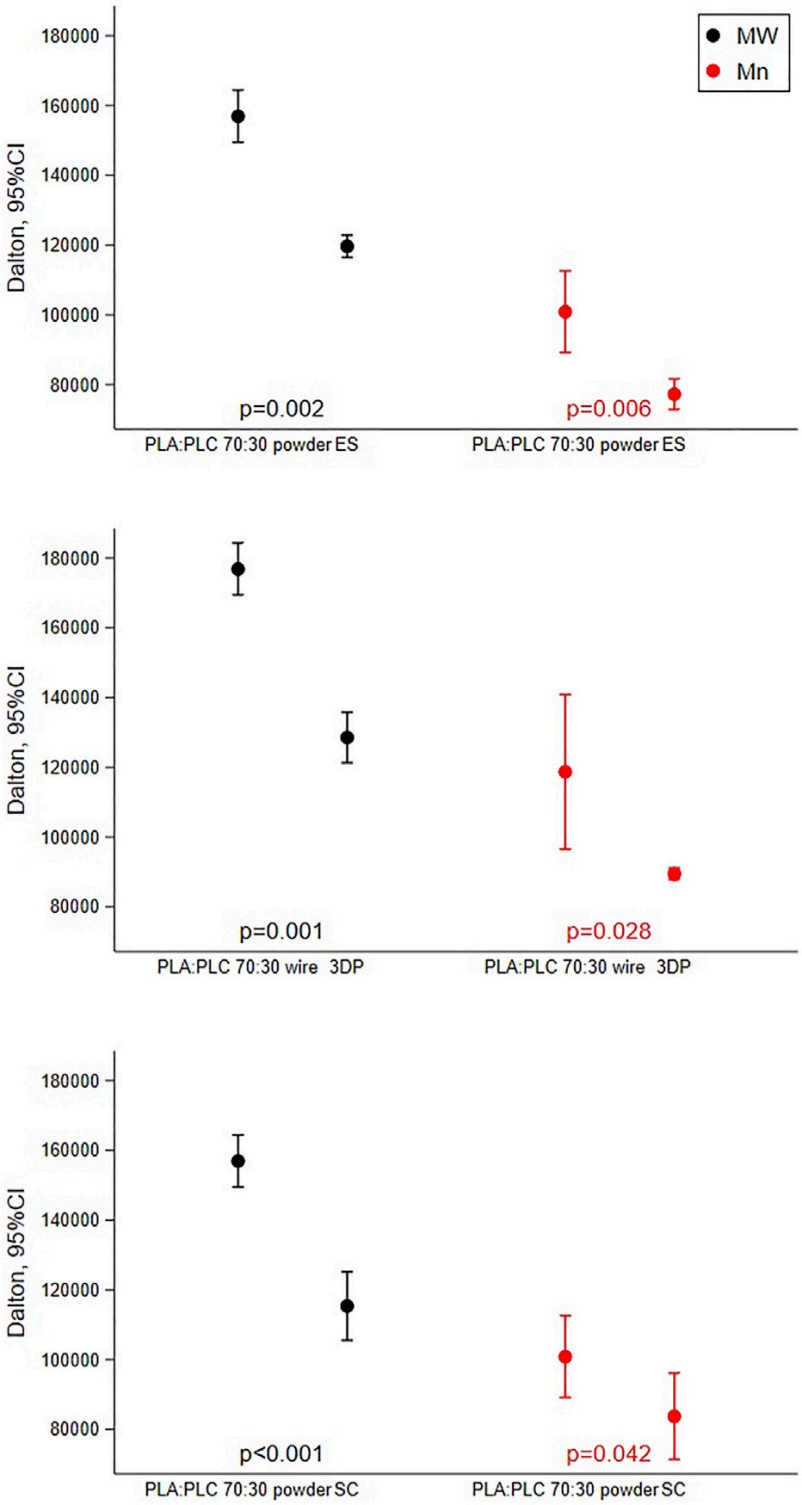
Results of GPC analysis reporting mean with 95%CI of molecular weight and molecular number performed on PLA-PCL 70:30 powder, wire, and scaffolds obtained by SC, ES, and 3DP.

Results obtained shown how all scaffold production techniques lead to a reduction in polymer molecular weight compared to the raw materials (Figure 2). In the case of SC and ES techniques, PLA-PCL powder was solubilized in solvents which were then evaporated to obtain the dry scaffolds, while in the case of 3DP the PLA-PCL wire was softened with heat (T > Tg°) to be extruded.

In all cases, between the raw material and the respective scaffold obtained there was a reduction in the molecular weight of 26% ± 3.7%.

### 3.3 Mechanical characterization

**FIGURE 3:**
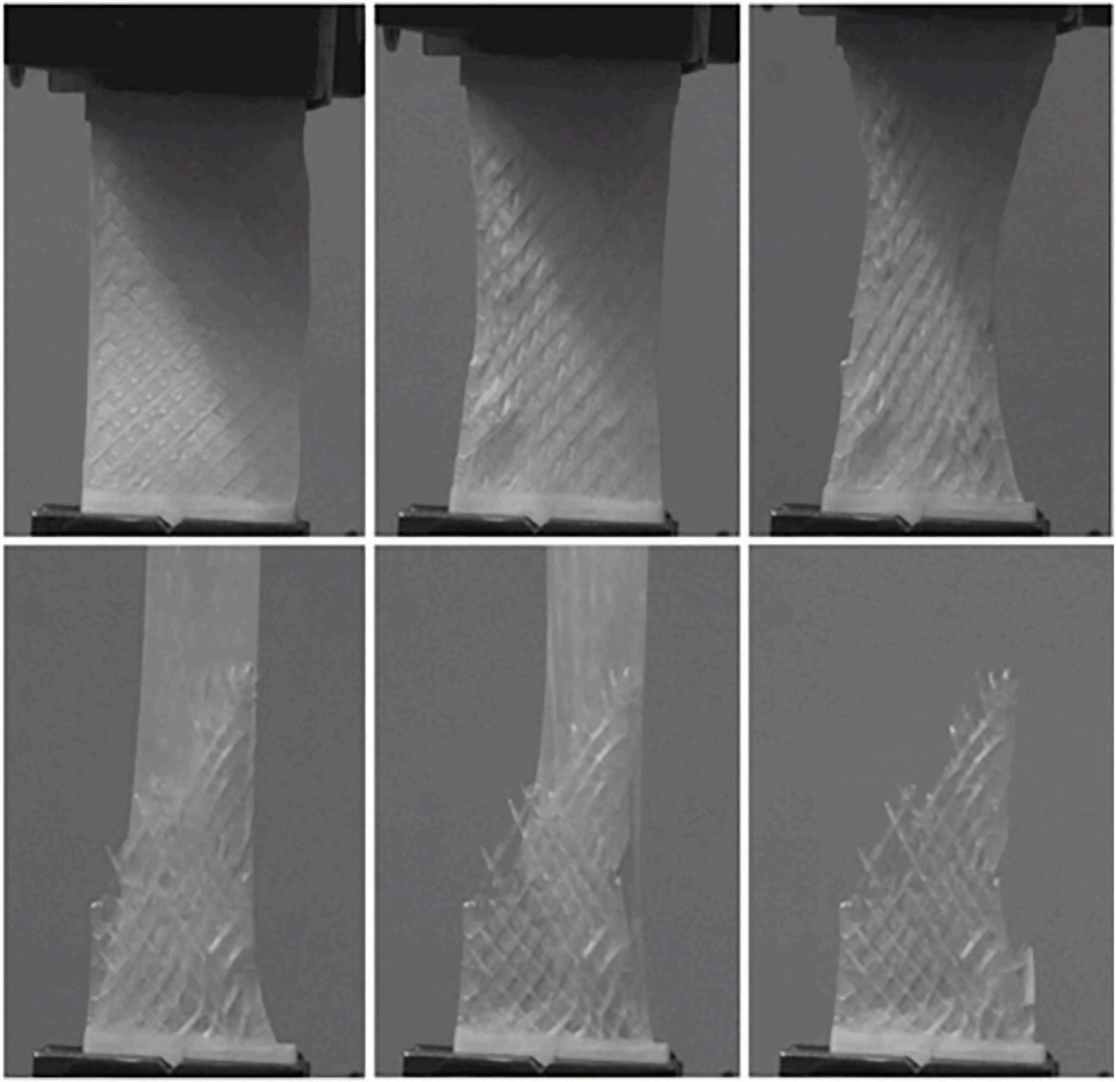
Different frames of uniaxial tensile test performed on HS scaffolds (20% infill).

**FIGURE 4:**
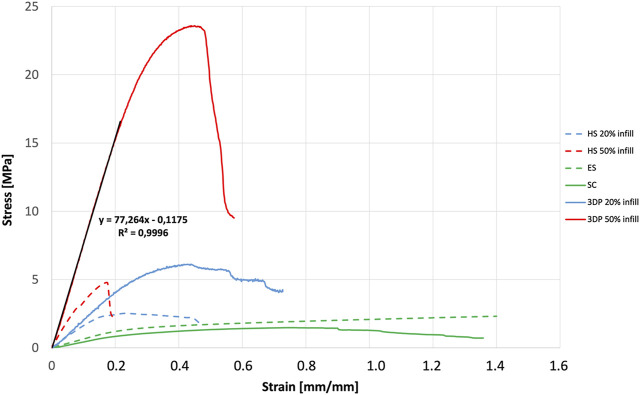
Mechanical characterization: stress-strain curves and slope resulting from the mechanical characterization of SC, ES, 3DP, and HS scaffolds.

Results obtained by uniaxial tensile tests were reported in Figures 3, 4. ES exhibited E = 6.21 ± 3.45 MPa while 3DP scaffolds showed higher values (E = 20.90 ± 6.11 MPa and E = 77.26 ± 23.58 MPa for the 20% infill and 50% infill scaffolds respectively). SC scaffolds showed lower E values (E = 4.56 ± 1.47 MPa), indicating that they are less stiff with respect to the previous ones.

HS 20% infill and HS 50% infill scaffolds showed values of E = 7.39 ± 3.85 MPa and E = 34.77 ± 6.37 MPa respectively. Stress-strain curve for 3DP 100% and HS 100% infill was not reported in the graph because their E values resulted out of scale if compared to the other ones (E > 100 MPa). These scaffolds resulted too rigid and stiff and – for these reasons – not suitable to mimic soft tissues’ mechanical properties.

The Young’s modulus values obtained from the tensile tests performed on the different scaffolds shown how even if the same material is used, the production technique influenced by the mechanical properties of the final structure. In fact, the production technique rules the resulting sub-millimeter microstructure (see Figure 1) which governs the mechanical response. All scaffolds have E values that fit within the physiological mechanical strength values reported in the literature for different soft (E ≈ 1 MPa) and hard tissues (E > 10 MPa) (Akhtar et al., 2011; Guimarães et al., 2020). Specifically, SC, ES, 3DP 20% infill, and its hybrid equivalent scaffolds can find their application in muscle and cartilage tissue regeneration. On the other hand, the 3DP 50% and 100% infill scaffolds are more suitable for bone regeneration, where much greater mechanical stiffness and strength is required.

### 3.4 Cell seeding and viability

**FIGURE 5:**
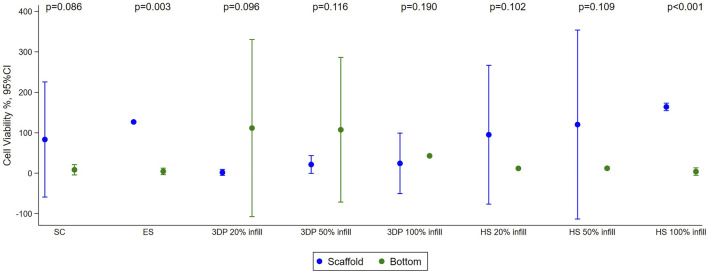
Results of cell viability test performed with HNDF (Human normal dermal fibroblast) 48 h after sowing on SC, ES, 3DP, and HS scaffolds. Mean with 95%CI are shown.

Results of cells seeding and viability % were reported in Figure 5.

The cell viability test showed good cell viability for cells seeded on SC (83% ± 21%) and ES (127% ± 15%) scaffolds. Almost no cells were found at the bottom of the multi-well, indicating that the scaffolds were able to retain the cells on their surface. The fact that the cell viability value of the ES scaffolds is higher than the control is justified as the adhesion and growth of the cells seeded on the scaffold and on the 2D control (multi-well plate) can be different due to the diversity of the support. In this case, there was better adhesion to the three-dimensional polymeric scaffolds than to the multi-well, justifying slightly higher vitality values. For the 3DP 20% infill and 50% infill scaffolds, most of the cells remained viable, but deposited at the bottom of the multi-well. This happened because the high porosity created by the geometry of the scaffolds does not retain the cells that fall by gravity and do not adhere to the scaffolds. The 3DP 100% infill scaffold demonstrated another problem; the absence of porosity and the smooth surface do not allow good cell adhesion (Pisani et al., 2020; Pisani et al., 2022). Increase in cell viability and adhesion was achieved in HS that exploit the porous texture of electrospun nanofibers combined to the rigidity of the 3DP scaffold. This is demonstrated to regulate cell behavior since substrate stiffness affects many different processes, such as cell growth, migration, and differentiation (Breuls et al., 2008).

**FIGURE 6:**
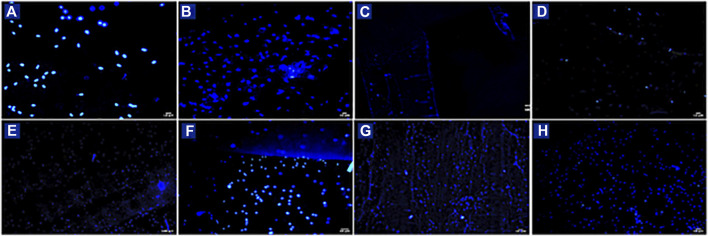
DAPI staining of HNDF 48 h after sowing on **(A)** SC; **(B)** ES; **(C)** 3DP 20% infill; **(D)** 3DP 50%; **(E)** 3DP 100% infill; **(F)** HS 20% infill; **(G)** HS 50% infill; and **(H)** HS 100% infill scaffolds.

**FIGURE 7:**
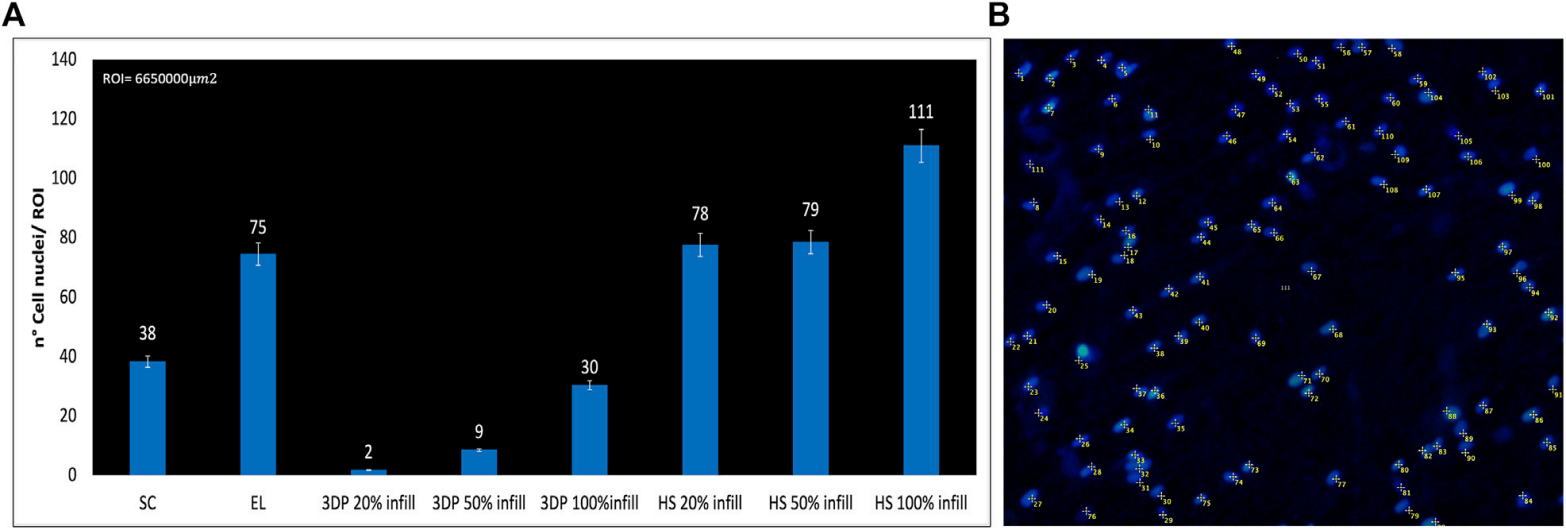
**(A)** Number of DAPI-stained cell nuclei for the region of interest (ROI); **(B)** Example of DAPI nucleus counting on HS 100% infill scaffolds.

Biological characterization was also performed by fluorescent microscopy through nuclear DAPI staining (Figures 6, 7). Images obtained reflect cell viability data in which a higher number of cells appears on ES scaffolds than on SC or 3DP scaffolds (both 20% and 50% infill). 3DP 100% infill scaffolds show some cells on the surface, but with few clusters. All the HSs showed a high cell density and complete cellularization of the electrospun surfaces with the initial formation of clusters.

**FIGURE 8:**
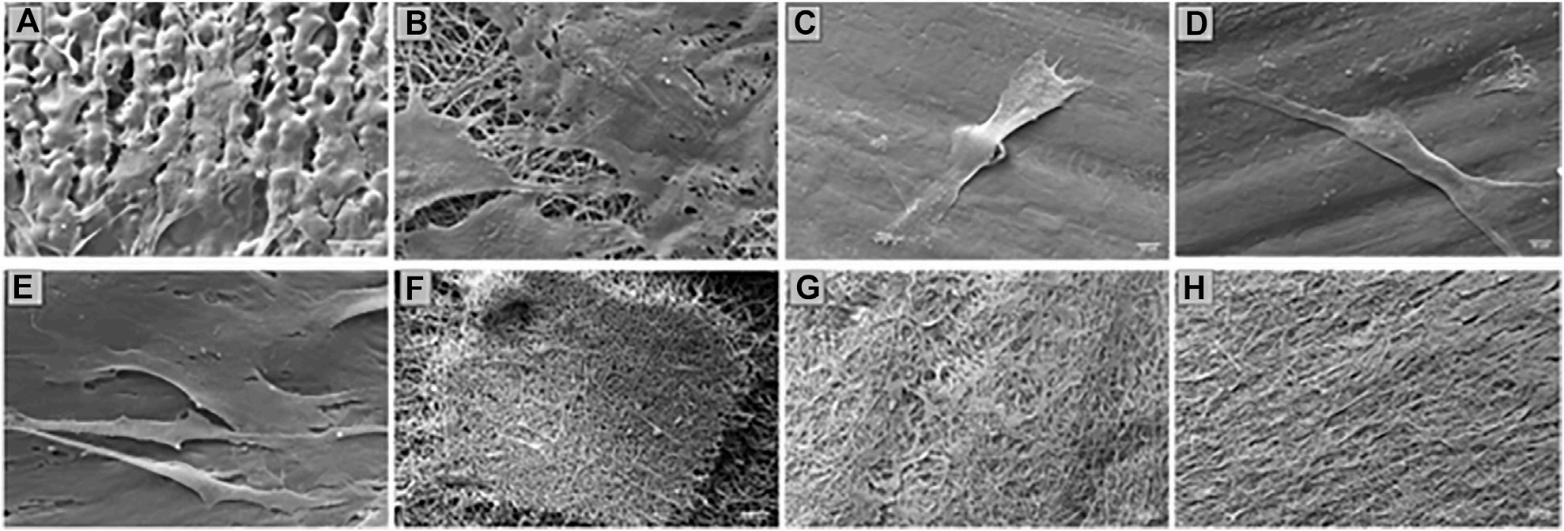
SEM of HNDF 48 h after sowing on **(A)** SC; **(B)** ES; **(C)** 3DP 20% infill; **(D)** 3DP 50% infill; **(E)** 3DP 100% infill; **(F)** HS 20% infill; **(G)** HS 50% infill; and **(H)** HS 100% infill scaffolds.

Confirmation of the presence of cells on the scaffolds was obtained from SEM images (Figure 8). Also, in this case there are more cells on the EL than on SC scaffolds. Only a few isolated cells were detected on 3DP 20% and 50% infill scaffolds. 3DP 100% infill scaffolds showed few living cells, which tend to detach from the scaffolds because of their smoother surface that avoids correct cell adhesion and proliferation. Otherwise, HSs show good surface cellularization with large spaces to allow further cell proliferation.

## 4 Discussion

The demonstrated improved performance of HSs produced through the combination of different techniques (EL and 3DP) is a step forward for current investigations. First of all–by analyzing the time-quality balance of the different production methods—it emerges that SC is a very long manufacturing technique requiring more than 24h, and not at all functional for the development of BSs due to scaffolds’ poor reproducibility and non-uniformity that compromise final outcomes. On the contrary, ES and 3DP techniques are characterized by short production times and a high process reproducibility. ES produces uniform and dimensionally homogeneous nanofibers which can be employed alone as scaffolds. Moreover, nanofibers offer the possibility to be used as a “functional coating material”, exploiting their high surface area over volume ratio to improve cell adhesion (Kishan and Cosgriff-Hernandez, 2017). 3DP technique—based on a defined CAD design—can be modified and adjusted in order to perfectly adapt to the user needs, offering high customization possibilities in the perspective of personalized precision medicine purposes (Vaz and Kumar, 2021). Moreover, important to consider is the scaffolds’ resolution that the different technologies can implement. The obtainable minimum thickness of scaffolds with the 3D technique at our disposal has been created to adapt to the values of mechanical properties compatible with soft tissue. On the other hand, the EL scaffolds, which mainly have a biological role in this case, as the nanofibrous texture improves cell adhesion, were electrospun for a suitable time to allow deposition both on the metal collector and on the polymeric 3D-printed scaffold.

Promising results have emerged from the integration of the two techniques: HS with different infill percentages (20% and 50%) show an improved surface porosity than the respective 3DP scaffolds. Particularly, in the case of the 100% 3DP scaffolds with no porosity, the addition of nanofibers leads to a suitable surface porosity able to guarantee cells’ adhesion. These values obtained for the HS scaffolds were more suitable to be used as supports for cell cultures and to allow the passage of nutrients/waste (Yadav et al., 2021). From the biological point of view, cell viability studies also demonstrated that HS scaffolds (20%, 50%, and 100% infill) show not only a higher viability than the respective 3DP and ES scaffolds, but also a better ability to retain cells on their surfaces. In fact, 3DP 20% and 50% scaffolds’ higher porosity did not allow the cells to adhere to the scaffold surface, making them fall to the multi-well bottom. On the other hand, the lack of porosity of 3DP 100% scaffolds did not guarantee suitable cell adhesion and growth, showing lower cell viability values. Both problems can be overcome by the combination between 3DP and ES, with the ES substrate improving the HS biological surface properties. It is also interesting to note that the performance of ES scaffolds is improved by the integration with 3DP support. It is therefore clear that HS scaffolds offer better biological performance than the ones produced through the two manufacturing techniques individually. Moreover, cell viability on HS scaffolds increased by increasing the infill of the 3DP scaffold. Moreover, when PLA-PCL was used to print the 100% 3DP scaffolds, the mechanical properties resulted were too high to be used as a support for soft tissue replacement and regeneration. Thus, an important aspect on which future research will focus will be to evaluate the use of less stiff materials for the development of high infill 3DP scaffolds for soft tissue regeneration.

From the biomechanical point of view, it was noted that the HS (20% and 50% infill) show lower Young’s modulus values (E) than the corresponding 3DP scaffolds: the high stiffness being one of the main problems of 3DP scaffolds, the integration of ES nanofibers offers the possibility to tune this parameter and overcome mechanical limitations. The measured values are compatible with the Young’s moduli values of some human soft tissues: in particular, possible applications for the proposed PLA-PCL HS could be the replacement of the aorta (2.0–3.0 MPa), myocardium (2.0–4.0 MPa), nerves (5 MPa), cartilage (5.7–6.2 MPa), and ligaments (25–93 MPa) (Guimarães et al., 2020; Kwon et al., 2020). Moreover, other studies highlighted that, especially in biological materials, mechanical properties are more controlled by the nano-micro-structure. At the nanostructure level of ECM proteins (10^−9^–10^−6^ m) stiffness is higher (10–10^3^ MPa) compared to the tissue (10^−5^–10^−3^ m) and organ level (10^−3^–10 m), which show a stiffness ranging from 10^−3^ and 10^2^ MPa (Akhtar et al., 2011). Because scaffolds also have to maintain mechanical strength and integrity at the nanoscale level unless new tissue regeneration takes place, it is acceptable that Young’s modulus of BSs is higher compared to the target tissue/organ (Yadav et al., 2021). However, for future applications, modulation of the E values could be adapted to the physiological values of other tissues/organs, their architecture, and individual needs, and modified according to the use of materials, architecture-geometry, and the presences of selected cells on the scaffold (Sultana, 2018; Pisani et al., 2022).

## 5 Conclusion

The proposed work stands as a “proof of concept” to validate the integrated use of two of the most innovative and scalable scaffolds’ production techniques, namely, electrospinning and 3D printing. Preliminary results obtained from this work defined some crucial aspects that are certainly very interesting for future development of more advanced BOs. HSs can be modulated as a function of i) used materials, ii) 3D construct micro-geometry, and iii) electrospinning nano-topography, to be adaptable to different soft tissue/organ types. BSs are therefore a valid option for further studies and advanced development of BOs could be used as alternatives to organ transplantation.

## Data Availability

The original contributions presented in the study are included in the article/Supplementary Material, further inquiries can be directed to the corresponding author.

## Abbreviations

MTT, 3-(4,5-Dymethiltiazol-2-Y)-2,5 Diphenyltetrazolium Bromide; 3DP, 3D Printing; DAPI, 4′,6-Diamidino-2-Phenylindole); BOs, Bioartificial Organs; BSs, Bioartificial Scaffolds; CAD, Computer Aided Design; DMSO, Dimethyl sulfoxide; ES, Electrospinning; EtOH, Ethanol; ECM, Extracellular Matrix; FFF, Fused Filament Fabrication; GPC, Gel Permeation Chromatography; Tg°, Glass Transition Temperature; HNDF, Human Normal Dermal Fibroblast; HS, Hybrid Scaffolds; L, Length; Mn, Molecular Number; Mw, Molecular Weight; PLA-PCL, Poli-L-Lactide-Co-Caprolactone; PDI, Polydispersity Index; SEM, Scanning Electron Microscopy; SC, Solvent Casting; THF, Tetrahydrofuran; T, Thickness; W, Width; E, Young’s Modulus.
